# Professor Helmut Acker (1939–2025): A Pioneer in Oxygen Sensing and Molecular Physiology

**DOI:** 10.1007/s00424-025-03122-3

**Published:** 2025-09-19

**Authors:** Till Acker, Joachim Fandrey, Jörgen Carlsson

**Affiliations:** 1 Institute of Neuropathology, University Hospital Giessen and Marburg GmbH, Arndtstraße 16, 35392 Gießen, Germany; 2 Institut für Physiologie, Universität Duisburg-Essen, Hufelandstraße 55, 45147 Essen, Germany; 3 Department of Immunology, Genetics and Pathology, Uppsala University, Rudbecklaboratoriet, 751 85 Uppsala, Sweden


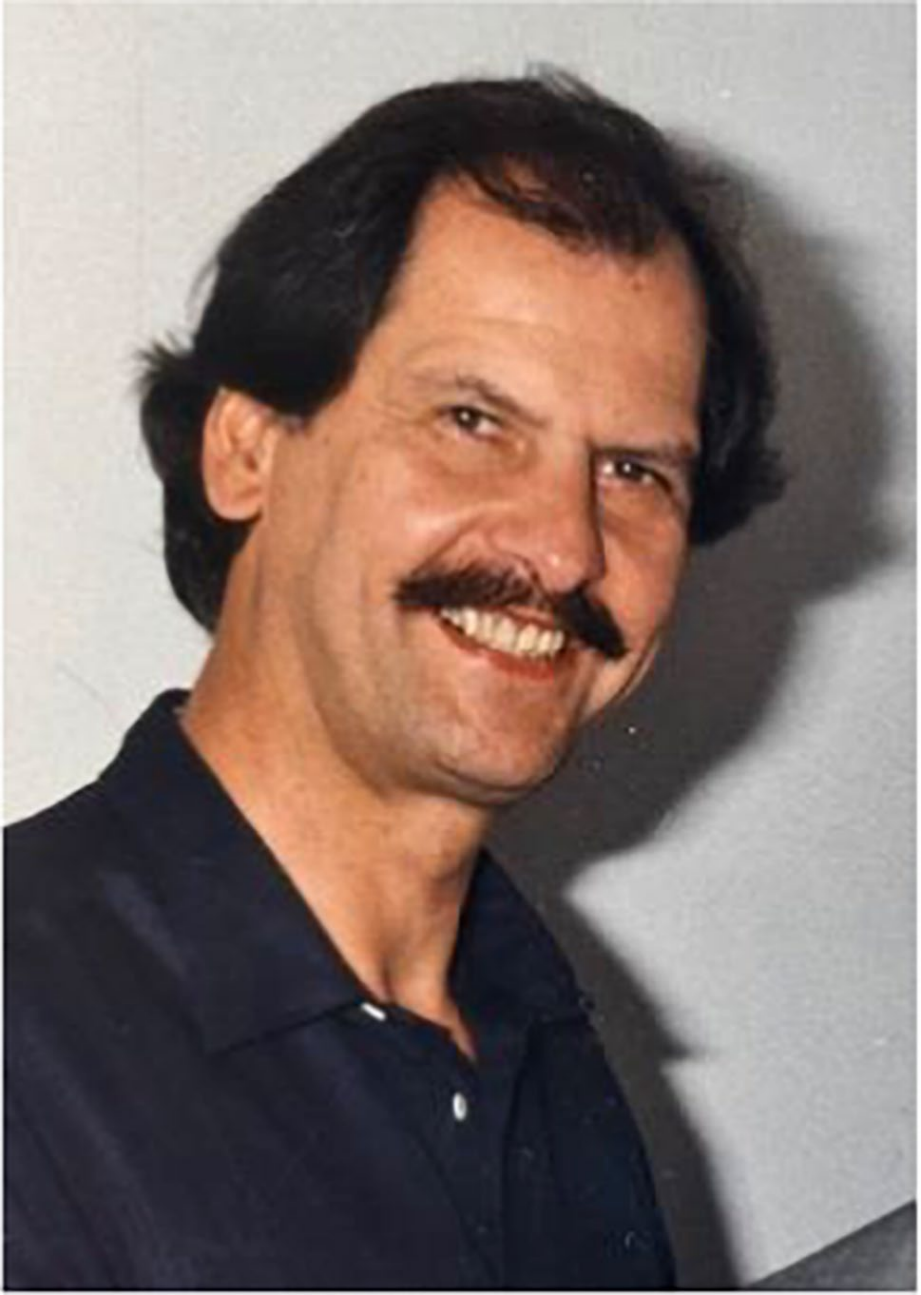


It is with great sadness that we announce the passing of Professor Helmut Acker, who died at the age 86 after a period of illness. Born on February 22, 1939, in Düren, Germany, he studied medicine at the Universities of Freiburg, Erlangen, and Innsbruck, graduating in 1965. He obtained his doctorate in 1967 and completed his habilitation in physiology at the Ruhr University Bochum in 1975.

Professor Acker spent the majority of his distinguished scientific career at the Max Planck Institute in Dortmund (later renamed the Max Planck Institute for Molecular Physiology), where he led the research group “Oxygen Sensing Physiology.” He also held appointments as Adjunct Professor at the Ruhr University Bochum and later as Guest Professor at the University of Duisburg-Essen, where he continued teaching and research well beyond his formal retirement in 2004.

His research focused on the cellular and molecular mechanisms of oxygen sensing and hypoxic signaling. He was a pioneer in the study of the carotid body and its role in chemoreception. Through groundbreaking work on mitochondrial function, redox biology, and ion channel physiology, he advanced our understanding of the hypoxia response in neural and tumor tissues. Largely through Helmut Acker’s pioneering work, microelectrode techniques became a widely recognized and internationally adopted method in physiological research.

Professor Acker’s scientific expertise encompassed a broad range of experimental approaches, including:

- Respiration physiology, especially carotid body physiology.
- Biology of endothelial cells and mitochondria in relation to oxygen metabolism.
- Oxygen dependent gene expression and ion channel conductivity.
- Hypoxia-inducible Factor (HIF-1) signalling.
- Oxygen and ion-sensitive microelectrodes for cells and tissue analysis.
- High resolution imaging by one-photon multifocal and two-photon confocal microscopy.
- Intracellular protein interaction and oxygen radical kinetics by FRET.
- Near Infrared Spectroscopy (NIRS)
- Three-dimensional tissue culture of tumour spheroids and organoids
- Physiology of embryonic stem cells.
- Acupuncture treatment of chronic pain and stress-related disorders.

Further details on these fields and a selection of Professor Acker’s publications can be accessed via the QR code below:
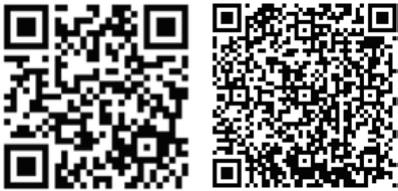


Helmut Acker was deeply committed to international collaboration, working regularly as a guest scientist and hosting numerous colleagues from abroad. One notable example was his pioneering contribution to tumour spheroid research. Collaborating with renowned scientists from Sweden, he investigated the physiological properties of non-vascularized micrometastases using three-dimensional tumour cultures. Helmut Acker is regarded as one of the key pioneers in this field. His work integrated physiological and biochemical perspectives and helped define the role of oxygen, pH, ion, and nutrient gradients in tumour biology — insights that anticipated the development of modern tumour organoid models and personalized oncology. These studies provided foundational knowledge about the biology of small, avascular metastases, particularly the mechanisms underlying proliferation, central necrosis, and variable therapeutic responses. His collaborative research helped establish tumour spheroids as a model system and contributed significantly to the conceptual framework of contemporary cancer research.

In his role as supervisor, Helmut Acker supported numerous researchers in completing their doctoral degrees. He organized several international meetings and scientific conferences. Over the course of his career, he published more than 160 scientific articles and authored or co-edited three influential books with Springer:

- Oxygen Sensing in Tissues. Helmut Acker, 1988 [1]
- Chemoreceptors & Chemoreceptor Reflexes. Acker et al. 1990 [2]
- Recent Results in Cancer Research; Spheroids in Cancer Research. Acker et al. 1984 [3]

He served as Secretary General of the International Society for Arterial Chemoreception (1987–1995) and was a trusted reviewer for national and international science foundations (including DFG, BMBF, Wellcome Trust, MRC, and NSF). His expertise was also sought in numerous collaborative research consortia and evaluation panels.

Helmut Acker remained scientifically active long after his retirement. With most of his exceptional 2-photon microscopy set-up, he moved to the Institute of Physiology at the University of Duisburg-Essen to continue his work on the mechanisms of oxygen sensing in the carotid body. Several publications such as in *The American Journal of Physiology* [4, 5] document these successful years. Moreover, Helmut Acker continued to teach with great enthusiasm. Until last year, he regularly gave seminars on carotid body physiology, introducing students to the physiology of oxygen sensing, His passion for the subject fascinated many – some of whom chose physiology for their doctoral thesis.

In addition, Helmut Acker remained a passionate advocate for science policy and academic development. Until shortly before his death, he served as Chair of the Scientific Board of the von Behring-Röntgen Foundation, where he was instrumental in shaping research funding strategies and mentoring early-career investigators in medicine and life sciences. In recognition of this longstanding service, he was awarded the von Behring-Röntgen Medal of Honour in 2024. Reflecting his broad intellectual curiosity, he also undertook scientific and clinical work in acupuncture — applying it in the treatment of chronic stress-related disorders and investigating its effects with the same critical rigour that marked his physiological research.

Helmut Acker will be remembered as a brilliant physiologist, a generous mentor, a devoted teacher and a visionary thinker whose work has left a lasting mark on biomedical science. He was also deeply committed to making scientific knowledge more widely accessible — a principle that shaped both his teaching and his research collaborations throughout his career.

Helmut Acker is mourned by his wife, two sons with their families, relatives, and a wide circle of colleagues, students, and friends. With his death, the scientific community has lost a unique creator of ideas and inspiration. We honour his legacy with deep appreciation and sincere gratitude.
